# Vasorelaxant Effects of *Syzygium samarangense* (Blume) Merr. and L.M.Perry Extract Are Mediated by NO/cGMP Pathway in Isolated Rat Thoracic Aorta

**DOI:** 10.3390/ph15111349

**Published:** 2022-10-31

**Authors:** Noura A. Hassan, Mohamed A. O. Abdelfattah, Yasmine M. Mandour, Assem M. El-Shazly, Mansour Sobeh, Mona F. Mahmoud

**Affiliations:** 1 Department of Pharmacology and Toxicology, Faculty of Pharmacy, Zagazig University, Zagazig 44519, Egypt; 2 College of Engineering and Technology, American University of the Middle East, Kuwait; 3 School of Life and Medical Sciences, University of Hertfordshire Hosted by Global Academic Foundation, New Administrative Capital, Cairo 11578, Egypt; 4 Department of Pharmacognosy, Faculty of Pharmacy, Zagazig University, Zagazig 44519, Egypt; 5 Faculty of Pharmacy, El Saleheya El Gadida University, El Saleheya El Gadida 44813, Egypt; 6 AgroBioSciences, Mohammed VI Polytechnic University, Lot 660-Hay Moulay Rachid, Ben-Guerir 43150, Morocco

**Keywords:** *Syzygium samarangense*, vasodilators, endothelium, nitric oxide, prostacyclin

## Abstract

*Syzygium samarangense* (Blume) Merr. and L.M.Perry is utilized widely in traditional medicine. We have reported previously a wide array of pharmacological properties of its leaf extract, among them anti-inflammatory, antioxidant, hepatoprotective, antidiabetic, antiulcer, and antitrypanosomal activities. We also annotated its chemical composition using LC-MS/MS. Here, we continue our investigations and evaluate the vasorelaxant effects of the leaf extract on aortic rings isolated from rats and explore the possible underlying mechanisms. *S. samarangense* extract induced a concentration dependent relaxation of the phenylephrine-precontracted aorta in the rat model. However, this effect disappeared upon removing the functional endothelium. Pretreating the aortic tissues either with propranolol or NG-nitro-L-arginine methyl ester inhibited the relaxation induced by the extract; however, atropine did not affect the extract-induced vasodilation. Meanwhile, adenylate cyclase inhibitor, MDL; specific guanylate cyclase inhibitor, ODQ; high extracellular KCl; and indomethacin as cyclooxygenase inhibitor inhibited the extract-induced vasodilation. On the other hand, incubation of *S. samarangense* extract with aortae sections having their intact endothelium pre-constricted using phenylephrine or KCl in media free of Ca^2+^ showed no effect on the constriction of the aortae vessels induced by Ca^2+^. Taken together, the present study suggests that *S. samarangense* extract dilates isolated aortic rings via endothelium-dependent nitric oxide (NO)/cGMP signaling. The observed biological effects could be attributed to its rich secondary metabolites. The specific mechanisms of the active ingredients of *S. samarangense* extract await further investigations.

## 1. Introduction

Vasodilators are commonly used to treat cardiovascular diseases and high blood pressure. In vasorelaxation, gaseous signalling molecules (gasotransmitters) such as nitric oxide (NO), carbon monoxide, and hydrogen sulphide play an important role [1]. The vascular endothelium is involved in the regulation of inflammation, platelet aggregation, and thrombosis, as well as influencing vascular tone and vasomotor function. The main mediator of these physiological processes is the endothelial derived relaxing factor, NO [2]. NO is formed utilising L-arginine as a substrate in a reaction catalysed by nitric oxide synthase (NOS). It activates soluble guanylyl cyclase (sGC), which enhances guanosine 3′, 5′-cyclic monophosphate (cGMP) synthesis indirectly. This activates protein kinase G, which reduces the sensitivity of a group of contractile elements to Ca^2+^ and suppresses Ca^2+^ inflow. Reduced NO bioavailability is commonly associated with endothelial dysfunction that may be an early stage of vasculopathy, which may develop to atherosclerotic cardiovascular disease [3].

In addition, Ca^2+^ ions influx via receptor-operated Ca^2+^ channels (ROCCs) and/or voltage-dependent Ca^2+^ channels (VDCCs) lead to contractile response in smooth muscle. Calcium channel antagonists, which are endothelium-independent vasodilators, have been shown to reduce the intracellular Ca^2+^ ions concentration in smooth muscle by inhibiting VDCCs resulting in a vasorelaxant effect [4]. Furthermore, K^+^ channels play a crucial function in the modulation of the vascular tone. The K^+^ ions channels in arteries do, in fact, change the resting membrane potential, which has an indirect effect on vascular tension. The vasodilator effects of many vascularly bioactive substances and medications are mediated by modulating K^+^ channels’ state [5]. Different chemical vasodilators such as calcium channel blockers, alpha-blockers, and direct vasodilators, which are used in managing hypertension and cardiovascular diseases, are costly and possess wide range of adverse effects. These side effects, together with low patient compliance and the high cost of the existing medications, prompted the search for new therapeutic options.

Traditional medicine makes extensive use of *Syzygium samarangense* (Blume) Merr. and L.M.Perry owing to its wide array of biological effects including antioxidant, anti-inflammatory, antiulcer, antitrypanosomal, hepatoprotective, and antidiabetic properties [6,7,8,9,10]. Previous studies showed that other species of *Syzygium* such as *S. guineense*, *S. polyanthum*, and *S. gratum* possessed vasorelaxant effects on isolated aorta and, accordingly, antihypertensive effects [11,12,13]. The current work studied the effect of *S. samarangense* extract on isolated aortae and tried to shed light on the possible underlying mechanisms.

## 2. Results

### 2.1. Chemical Composition

**Figure 1:**
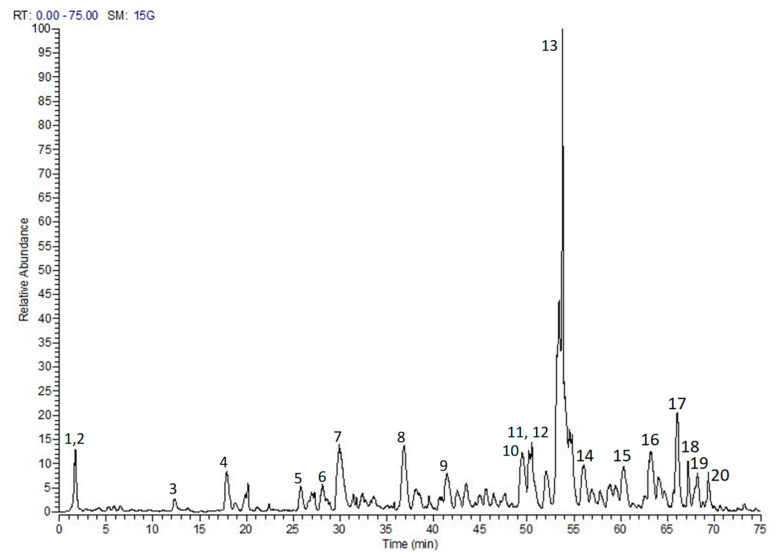
LC-MS/MS profile of *S. samarangense* leaves extract, adopted from Sobeh et al. [10]. (1) Quinic acid, (2) Hydroxyferuloyl malic acid, (3 and 4) (epi).Catechin-(epi)-gallocatechin, (5) (epi)-Gallocatechin-(epi)-catechin gallate, (6) Kaempferol glucoside, (7) (epi)-Gallocatechin gallate, (8) (epi)-Catechin-afzelechin, (9) (epi)-Catechin gallate, (10) Myricetin glucoside, (11) Myricetin pentoside, (12) (epi)-Catechin-(epi)-catechin-(epi)-catechin-(epi)-gallocatechin, (13) Myricitrin, (14) Procyanidin dimer monogallate, (15) Methyltricin, (16) Guaijaverin, (17) Isorhamnetin rhamnoside, (18) Mearnsitrin, (19) Myrigalone H pentoside, and (20) Quercetin galloyl-pentoside.

The phytochemical composition of the tested extract was previously annotated via LC-MS/MS. The analysis furnished 92 compounds including phenolic acids, flavonoids, tannins, and ellagitannins [10]. In another study, two secondary metabolites, namely myricitrin and 3,5-di-*O*-methyl gossypetin, were isolated and characterized via H- and ^13^C-NMR. Figure 1 shows LC-MS chromatogram of *S. samarangense* extract [9].

### 2.2. Vasodilatation Effect of S. samarangense Extract in the Endothelium-Intact Aorta Sections

**Figure 2:**
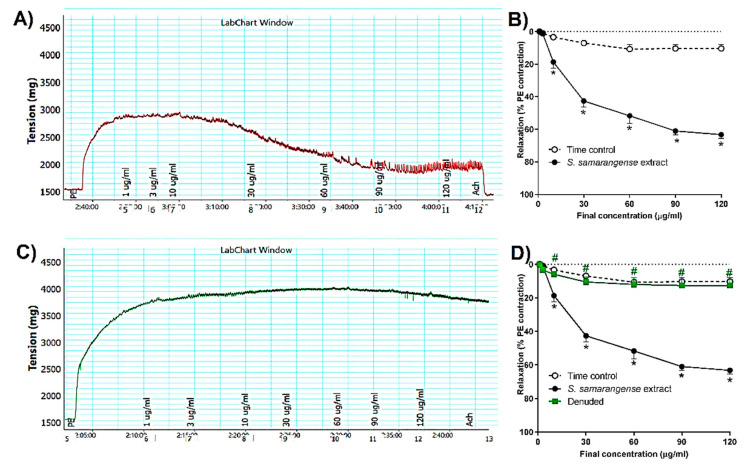
Effects of the cumulative addition of *S. samarangense* extract doses (1–120 g/mL) on isolated aortae from control rats that had been pre-constricted by phenylephrine (PE, 10 M). (**A**) typical tracing of *S. samarangense* extract’s dilation-inducing effects on PE-preconstricted aortae with healthy endothelium, (**B**) concentration response curve showing the vasodilating effect of *S. samarangense* extract on PE preconstricted aortae with intact endothelium compared with appropriate time controls, (**C**) an illustration of the effect of *S. samarangense* extract’s vasodilator on PE-precontracted aortae with denuded endothelium, and (**D**) concentration response curve showing the vasodilating effect of *S. samarangense* extract on PE preconstricted aortae with denuded endothelium compared with its vasodilating effect on PE pre-constricted aortae with the intact endothelium. The results are displayed as the mean ± standard error of six animals. In comparison to the time control values, * *p* ˂ 0.05; in comparison to the effect of the S. samarangense extract on intact endothelium values, # *p* ˂ 0.05.

Figure 2A,B represents the cumulative relaxation response curves to *S. samarangense* extract in rat aortae with intact endothelium isolated from control animals, where *S. samarangense* extract produced a significant vasodilation in the aortae vessels starting from a concentration of 10 μg/mL up to 120 μg/mL when compared to time-control values (all at *p* < 0.05).

### 2.3. Role of Endothelium Denudation in the Observed Vasodilation Effect of S. samarangense Extract

The vasodilation activity of *S. samarangense* extract was studied using aortae sections after removing the endothelium. The results show that *S. samarangense* extract induced vasodilation was completely inhibited by endothelium denudation at concentrations 10–120 ug/mL (all at *p* < 0.05, Figure 2C,D).

### 2.4. Role of Endothelial NO/cGMP Pathway in the Observed Vasodilation Effect of S. samarangense Extract

**Figure 3:**
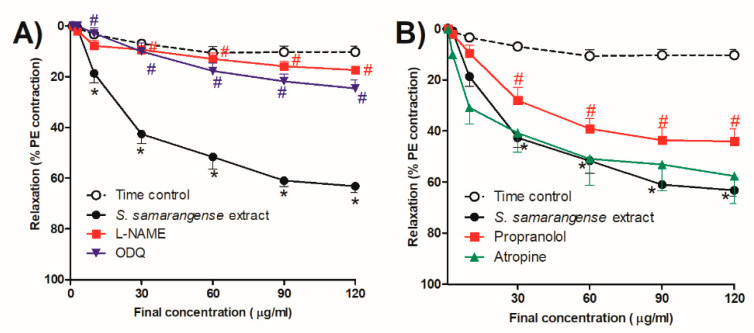
Effects of the cumulative addition of *S. samarangense* extract doses (1–120 g/mL) on isolated aortae from control rats that had been pre-constricted by phenylephrine (PE, 10 M). The effect of preincubation (30 min) with (**A**) Nω-nitro-L-arginine methyl ester hydrochloride (L-NAME, 100 μM)as a NO synthase inhibitor, and 1H-(1,2,4)-oxadiazolo(4,3-a)quinoxalin-1-one (ODQ, 1 mM) as a guanylate cyclase inhibitor, (**B**) Propranolol (1 µM) as a β-adrenoreceptor antagonist, and atropine (100 µM) as a standard muscarinic receptor blocker, on the vasodilation effect of *S. samarangense* extract on PE preconstricted aorta sections. The results are displayed as the mean ± standard error of six animals. * *p* < 0.05, compared with the time control values, # *p* < 0.05, compared with *S. samarangense* extract effect on intact endothelium values.

To investigate the role of NO dependent pathway in *S. samarangense* extract vasodilation, we evaluated the activity of the extract in the presence of the NO synthase inhibitor (L-NAME, 100 µM), which significantly inhibited *S. samarangense* extract (10–120 µg/mL)-induced vasodilation of the isolated endothelium-intact rat aorta (*p* < 0.05, Figure 3A). Moreover, the specific sGC inhibitor, ODQ (10 µM), caused significant decline in the vasorelaxing effect of the extract at concentrations 10–120 µg/mL (*p* < 0.05, Figure 3A).

### 2.5. Receptors Related to the Vasodilation Effect of S. samarangense Extract

We examined the effect of the extract in the presence of the β-adrenoceptor antagonist propranolol and the muscarinic antagonist atropine in order to investigate the potential receptor(s) involved in the extract’s vasodilation effect. At concentrations of 30–120 g/mL, propranolol significantly decreased the vasodilatation effect of *S. samarangense* extract. (*p* < 0.05, Figure 3B), while atropine did not affect significantly the vasodilation effect of *S. samarangense* extract (Figure 3B).

### 2.6. Investigation of the Potential Role of Endothelium-Dependent Hyperpolarization

**Figure 4:**
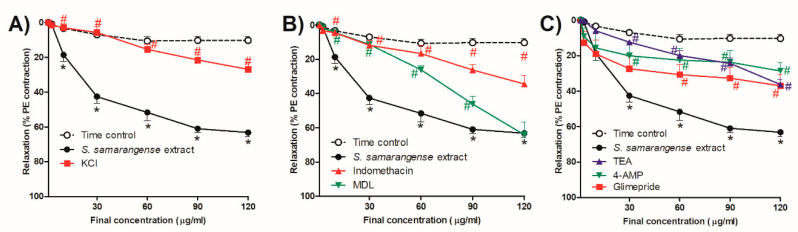
Effects of the cumulative addition of *S. samarangense* extract doses (1–120 g/mL) on isolated aortas from control animals that had been preconstricted by phenylephrine (PE, 10 M). The effect of preincubation (30 min) with (**A**) KCl (30 mM), as a membrane hyperpolarization inhibitor; (**B**) Indomethacin (INDO, 10 M), an inhibitor of cyclooxygenase; and cis-N-(2-Phenylcyclopentyl) azacyclotridec-1-en-2-amine, as an adenylate cyclase inhibitor. (**C**) Tetraethylammonium chloride (TEA, 10 mM), as a blocker of the standard voltage-dependent K^+^ channels, HCl (MDL, 30 M), the standard Ca^2+^-gated K^+^ channel blocker (4-AP, 1 mM), and glimepiride (10 µM) as a Sarc K^+^ ATP channel blocker on the vasodilation effect of *S. samarangense* extract on PE preconstricted aorta sections. The results are displayed as the mean± standard error of six animals. By using a two-way ANOVA and the Bonferroni post hoc test, the results were statistically significant when compared to the time control values, * *p* < 0.05 and the effect of the *S. samarangense* extract on intact endothelium # *p* < 0.05, respectively.

Figure 4A shows that inhibiting hyperpolarization by high extracellular KCl significantly reduced the vasodilation effect of *S. samarangense* extract at concentrations 10–120 µg/mL (all at *p* < 0.05).

### 2.7. Investigation of the Potential Role of Prostacyclin (PGI2)/cAMP Pathway in S. samarangense Extract-Induced Vascular Relaxation

Figure 4B shows that a cyclooxygenase inhibitor, indomethacin (10 µM), showed a significant inhibitory effect on the vasodilation action of *S. samarangense* extract at concentrations 10–120 µg/mL (*p* < 0.05, Figure 4A). However, AC inhibitor MDL (30 µM) significantly inhibited *S. samarangense* extract vasodilation activity at concentrations 10–90 µg/mL (*p* < 0.05, Figure 4B), where the inhibitory action of blocking AC on the vasodilation activity of the extract disappeared by increasing the extract’s concentration to 120 µg/mL.

### 2.8. Role of Different K^+^ Channels in the Observed Vasodilation Effect of S. samarangense Extract

As shown in Figure 4C, at concentrations between 30 and 120 g/mL, both 4-AP (1 mM), a voltage-dependent potassium channel blocker, and TEA (10 mM), a common Ca^2+^-gated K^+^ channels blocker, attenuated the vasodilating effects of the *S. samarangense* extract (*p <* 0.05). However, at concentrations between 60 and 120 g/mL, the ATP-sensitive potassium channel blocker glimepiride (10 M) greatly decreased the extract’s ability to dilate blood vessels. (All at *p* < 0.05, Figure 4C).

### 2.9. Role of Ca^2+^ Influx and Mobilization in S. samarangense Extract Induced Vasodilation

**Figure 5:**
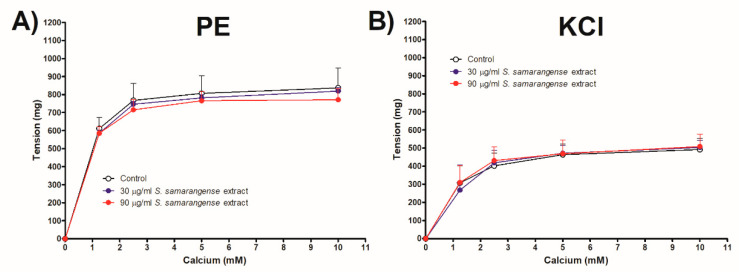
Dose response curve of CaCl_2_ in a Ca^2+^-free Krebs solution in the presence or absence of various concentrations of *S. samarangense* extract (30 and 90 g/mL) (**A**) in phenylephrine (10 μM) and (**B**) in KCl (80 mM)- induced constriction of endothelium-intact aortic rings, respectively. Results are displayed as the mean ± standard error of six animals.

Figure 5A,B demonstrates that the Ca^2+^-induced constriction of aortae vessels was unaffected by the incubation of *S. samarangense* extract (30 and 90 g/mL) with intact aortae sections that had been pre-constricted by either PE or KCl.

### 2.10. Molecular Docking

The in vitro results showed that the vasodilatory effects of *S. samarangense* extract are more likely mediated by the NO/cGMP pathway. NO exerts its pharmacological effects by binding to sGC receptors, which is recently validated as a main drug target for the cardiovascular diseases. Thus, we utilized the molecular docking tool to investigate the binding potential of the major compounds identified previously in the extract towards sGC receptor. Table 1 shows the docking scores (kcal/mol) of the compounds docked into sGC receptor in both activated and inactivated states. Noteworthy, several compounds were also explored; however, they failed to show any interactions with both states (activated and inactivated). These include thymol, phloretic acid, kaempferol, naringenin 7-methyl ether, scutellarein 6-methyl ether, gingerol, ellagic acid, pinoresinol, todolactol, and oleuropein aglycone.

**Figure 6:**
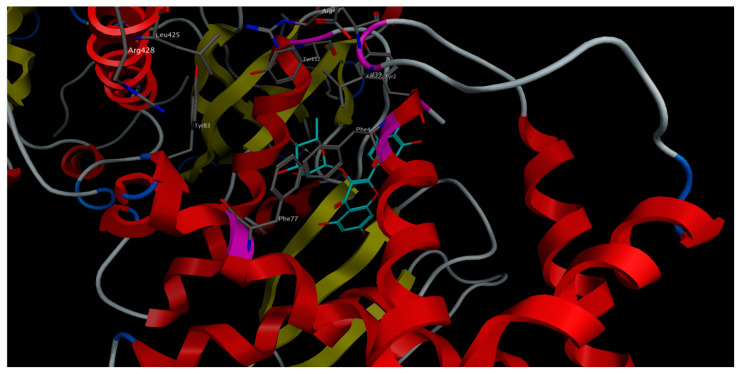
3D-interactions of myricitrin upon docking into the stimulators binding site in sGC-activated receptor state.

In general, the majority of *S. samarangense* extract compounds showed to fit properly in the sGC binding site in both activated and inactivated states. However, the docked compounds showed better binding affinity (lower docking score) to the receptor’s activated state rather than the inactivated state, indicated by the docking score values. Compounds showed a docking score range of −14.77 to −8.08 versus −12.53 to −6.83 kcal/mol on the receptor’s activated and inactivated states, respectively. Myricitrin achieved the best binding affinity to the sGC receptor in the activated state, showing the lowest docking score (−14.77 kcal/mol). It afforded different polar and non-polar interactions with the amino acid residues framing the binding site of the sGC stimulators in the sGC-activated state such as Phe4 (Figure 6).

**Figure 7:**
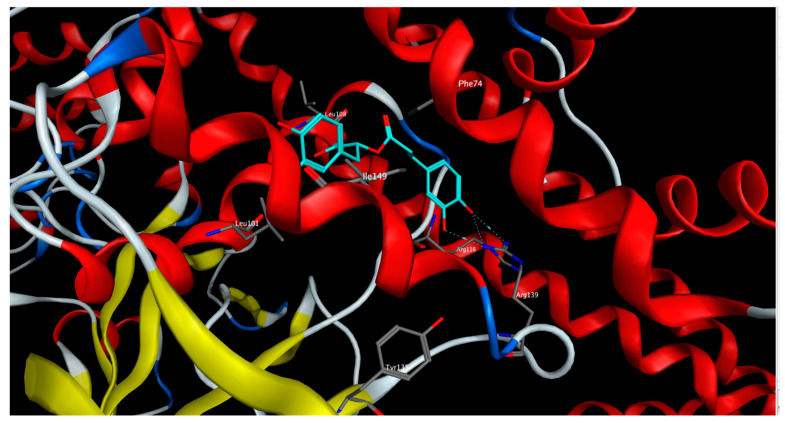
3D-interactions of rosmarinic acid upon docking into the activators binding site in sGC-inactivated receptor state.

On the other side, rosmarinic acid showed the lowest docking score (−12.53 kcal/mol) and the best binding affinity to the sGC receptor in the inactivated state, affording different interactions with the amino acid residues characterizing the binding site of the sGC activators in the sGC-inactivated state such as Leu101, Arg116, Arg139, and Ile149 (Figure 7).

## 3. Discussion

The current work is the first attempt to examine the in vitro vasodilatory properties of *S. samarangense* extract using isolated rat aortae. The isolated aortae technique is a widely used experimental model to identify the presence of the vasoactive substances in plant extracts and their metabolites and to validate their therapeutic potential in the management of hypertension [14]. Moreover, the isolated rat aorta model can be utilised to detect the different mechanisms by which these vasoactive substances can manage hypertension [15].

In the present study, we showed that *S. samarangense* extract (10–120 g/mL) has a concentration-dependent vasodilation effect on rat aortic rings that have been pre-constricted by PE. The vasorelaxant effect of *S. samarangense* extract is entirely endothelium dependent as mechanical denudation of the endothelium completely abolished the vasorelaxant effect of *S. samarangense* extract. To determine the exact mechanism by which *S. samarangense* extract exerted the endothelium-dependent vascular relaxation, we examined its relaxation effect in presence of different blockers of the candidate pathways.

In the cardiovascular system, the endothelium contributes to the maintenance of vasodilator tone by releasing endothelium-derived vasodilator factors namely NO, endothelium-derived hyperpolarizing factor (EDHF), and prostacyclin (PGI2) [16]. The observed vasorelaxant action of *S. samarangense* extract was investigated to establish the role of the NO/sGC/cGMP pathway. Therefore, the vessels were pretreated with L-NAME, a non-specific eNOS inhibitor, which completely abolished *S. samarangense* extract vasorelaxant effect. As soluble guanylate cyclase (sGC) is the primary sensor of NO [17], we utilized ODQ, a selective guanylate cyclase inhibitor, which strongly reduced the extract induced vasodilation.

Biochemical and structural studies revealed that sGC is a heterodimer of α and β subunits. The later harbors a ferrous haem, which is crucial for NO sensing. It was found out that haem oxidation and/or dissociation can trap the receptor in an inactive state that responds poorly to NO and has a different bent shape than the active state. The so far discovered sGC modulators are either stimulators or activators. The stimulator drugs, such as riociguat, can stimulate the activated state (haem is retained) of the receptors in presence or absence of NO, while activators, such as cinaciguat, can activate the receptors when present in an inactivated state (oxidized or dissociated haem), where NO signaling is impaired [18]. Molecular docking was utilized to gain some insights about the potential of *S. samarangense* extract’s major constituents to bind to sGC in its both states. We found that 9 compounds were able to bind the activated-sGC only, 6 compounds bound to the inactivated-sGC only, while 14 compounds were able to fit into both states but with better binding affinity to the activated receptor state (Table 1). In view of these findings, we can conclude that the docked compounds are more likely to act as sGC stimulators. This could be substantiated by the fact that these compounds have high antioxidant properties that would secure the ferrous form of the haem and hence an activated receptor state. Moreover, the in vitro results showed that the extract could not exert its vasorelaxant effects when NO is diminished by the NO synthase inhibitor, L-NAME, which means that the extract’s components are not likely to act as sGC activators. Taking together both in vitro and in silico results, we can suggest that the NO/sGC/cGMP pathway plays an essential role in *S. samarangense* extract mediating vasodilation. Further research is still required to learn better about the mechanism of the extract’s action.

Different receptors are involved in the process of activation of eNOS, such as cholinergic muscarinic receptors of the endothelial membrane which was found to activate eNOS to produce NO through increasing the intracellular concentration of Ca^2+^ ions [19,20]. Additionally, β_2_-adrenoceptor is involved in the regulation of eNOS activity and consequently vascular tone, by means of the protein kinase B dependent pathway [21]. Therefore, we performed a series of experiments using atropine, a muscarinic receptor antagonist, and propranolol, a β-adrenoceptor antagonist. We found that *S. samarangense* extract induced vasodilation was not affected by incubation with atropine but was significantly reduced in presence of propranolol. These results support concluding that cholinergic muscarinic receptor is not involved in the activation of eNOS induced by *S. samarangense* extract; however, activation of β-adrenoceptor seems to be involved in the activation of eNOS by *S. samarangense* extract.

We examined whether *S. samarangense* extract has the ability to induce releasing EDHF along with NO in muscular vessels as we performed a series of experiments in the presence of high extracellular K^+^ (30 mM), which reduces the electrochemical gradient for K^+^ and inhibits the EDHF response [22,23]. Our finding revealed that vascular relaxation to *S. samarangense* extract was attenuated by inhibiting EDHF, indicating the ability of the bioactive compounds of *S. samarangense* extract to stimulate the release of EDHF from the endothelium and producing vasorelaxation.

The role of PGI2, a potent vasodilator substance, in the observed vasodilation effect of *S. samarangense* extract was investigated as well in this work. The vasodilating activities of PGI2 are mediated through either activation of AC in VSMCs or through the release of EDHF that diffuses to and activates VSMC K^+^ channels, causing hyperpolarization [24,25]. In the present study, *S. samarangense* extract-induced relaxation was significantly attenuated by indomethacin. Moreover, we utilized the AC inhibitor, MDL, which produced temporary inhibition of the extract-induced vasodilation at dose range 10–90 µg/mL, but by boosting the *S. samarangense* extract concentration to 120 g/mL, this inhibition was overcome. We also showed that the PGI2/AC/cAMP pathway plays a role in mediating *S. samarangense* extract’s vasodilating action at low and medium but not high concentrations of *S. samarangense* extract. Meanwhile, indomethacin and high extracellular K^+^ (30 mM) significantly reduced *S. samarangense* extract-induced vasorelaxation at higher concentrations (120 µg/mL) indicating that PGI2 has a role in mediating vasodilation produced by higher concentrations of *S. samarangense* extract through stimulating the release of EDHF but not through activation of the AC/cAMP dependent pathway.

The activation of K^+^ channels participates, largely, in the regulation of the vascular tone, as they are the main determinant of the cell membrane potential. Endothelium-dependent vasodilators, NO, EDHF, and PGI2, activate K^+^ ions channels in the membrane, which in turn allows K^+^ ions efflux, leading to a reduction in the membrane potential and hyperpolarization and, as a consequence, closing voltage-gated Ca^2+^ channels in the cell membrane resulting finally in vascular muscle relaxation [26]. To study the possible involvement of K^+^ channels in the observed vasorelaxation effect of *S. samarangense* extract, we performed a set of experiments in the presence of different K^+^ channels blockers, namely TEA, a blocker of the nonselective Ca^2+^-gated K^+^ channel [27]; glimepiride, a blocker of the Sarc K^+^ ATP channels [28]; and 4-AP, a blocker of the voltage-dependent K^+^ channel [29]. All the tested K^+^ channel blockers reduced the vasorelaxant effect evoked by *S. samarangense* extract, pointing out the involvement of different types of K^+^ channels activation in the vasorelaxant effect of *S. samarangense* extract.

**Figure 8:**
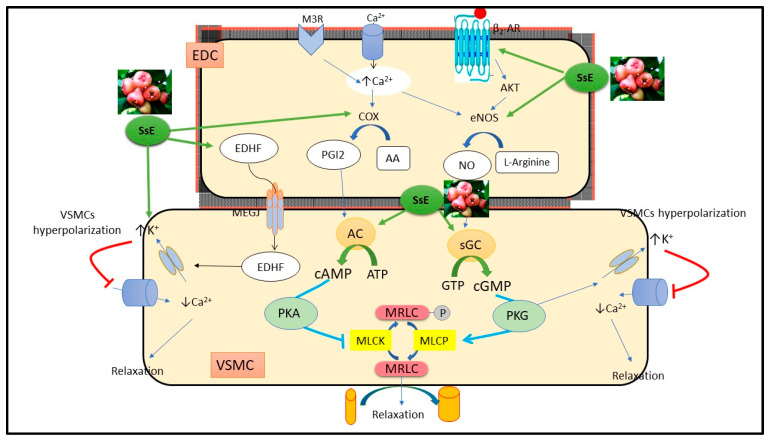
Mechanistic insight of *S. samarangense* extract on vascular reactivity of isolated aorta of rats. Abbreviations: AA; arachidonic acid; AC, adenylate cyclase; AKT, Protein kinase B; ATP, Adenosine triphosphate; β2-AR, β2 adrenoceptor; Ca^2+^, calcium; cAMP, cyclic adenosine monophosphate; c-GMP, cyclic guanosine monophosphate; COX; cyclooxygenase enzyme; EDC, endothelial cell; EDHF, endothelial dependent hyperpolarizing factor; eNOS, endothelial nitric oxide synthase; NO, nitric oxide; K^+^, potassium; PKA, protein kinase A; M3R, muscarinic 3 receptor; MEGJ, myoendothelial gap junction; MLCK, myosin light chain kinase; MLCP, myosin light chain phosphatase; MRLC, myosin regulatory light chain; P, phosphorous; PGI2, prostacyclin; VSMC, vascular smooth muscle cell; sGC, soluble guanylate cyclase; GTP, guanosine triphosphate; PKG, protein kinase G; SsE, *S. samarangense* extract. Adopted from Laban et al. [31].

The impact of *S. samarangense* extract on Ca^2+^ influx through voltage-dependent or receptor-operated Ca^2+^ channels [30] was also studied in the present study using Ca^2+^ free media. *S. samarangense* extract (30 and 90 µg/mL) did not alter the Ca^2+^ induced concentration dependent constrictions in isolated aortae preconstricted with either PE or KCl. These results indicate that *S. samarangense* extract has no blocking activity on Ca^2+^ influx via both receptor operated Ca^2+^ channels and voltage dependent Ca^2+^ channels. A detailed mechanistic insight is shown in Figure 8.

Noteworthy, many previous studies showed that phenolic compounds that constitute the major secondary metabolites in *S. samarangense* have promising effects on the smooth muscles from different organs [32,33].

## 4. Materials and Methods

### 4.1. Extraction, Drugs and Chemicals

*Syzygium samarangense* (Blume) Merr. and L.M.Perry leaves were collected and extracted as previously described [10]. The chemicals used in the current study included ketamine (Sigma pharmaceutical industries, Menoufia, Egypt), propranolol, 4- aminopyridine (4-AP), glimepiride (LKT Laboratories, Inc., St. Paul, MN, USA), phenylephrine (PE), tetraethylammonium chloride (TEA), Nω-nitro-L-arginine methyl ester (L-NAME), 1H-(1,2,4)-oxadiazolo(4,3-a)quinoxalin-1-one (ODQ), atropine, indomethacin, acetylcholine (Ach), cis-N-(2-Phenylcyclopentyl) azacyclotridec-1-en-2-amine HCl (MDL), and dimethyl sulfoxide (DMSO) (Sigma-Aldrich, Munich, Germany).

### 4.2. Animals

Male Wistar rats aged eight-week-old, with a weight of 180–200 g, were obtained from the animal house facility in the faculty of Veterinary Medicine, Zagazig University. Each four rats were housed in polypropylene clear cages with good ventilation. Temperature of 22 ± 2 °C, relative humidity of 50–60% with 12-h day, and night cycle were maintained. Rats were given free access to purified water and rodent pellet food. Animal handling procedures and the experimental design are in accordance with the guidelines approved by the Ethical Committee for Animal Handling (approval number; ZU-IACUC/3/F/73/2020) at Zagazig University.

### 4.3. Aortae Isolation

Aortae sections were prepared following the procedure in [34,35,36,37]. Rats were anesthetized with ketamine (100 mg/kg, i.p.) and had cervical dislocation for euthanasia. The thoracic cage was then opened, and the descending thoracic aorta was carefully isolated and placed in cold Krebs–Henseleit solution containing (in mM): NaCl 118.1, KCl 4.69, KH_2_PO_4_ 1.2, NaHCO_3_ 25.0, glucose 11.7, MgSO_4_ 0.5, and CaCl_2_ 2.5, and aerated with 95% O_2_ and 5% CO_2_. Connective tissues and fats were cleaned from the aorta then the aorta was cut into several rings each about 3 mm long. The rings were maintained under a resting tension of 1.5 g for 60 min.

### 4.4. Studying the Vasodilation Effect of S. samarangense Extract

The vasodilation potential of *S. samarangense* extract was studied using the isolated artery techniques mentioned previously in [34]. In brief, after maintaining the rings under equilibrium for 60 min, 10 µM of phenylephrine (PE) was used to induce vasoconstriction. The functional endothelium presence was affirmed if aorta sections produced relaxation more than 75% after adding acetylcholine (Ach, 10^−5^ M). Washout was applied and the tension returned to the basal levels. The isolated aorta sections were again constricted with 10 µM PE, then cumulative concentrations of *S. samarangense* extract in DMSO (1–120 µg/mL) were introduced to the baths. Before adding the next concentration, the aortae were allowed to reach relaxation plateau. The vehicle was added to time control channels to offset any possible effect by DMSO. The overall concentration of DMSO was not more than 0.1% in the organ bath. Just before terminating the experiment, a single dose of Ach (10 µM) was added to all the channels for a complete relaxation of aortae. To investigate the role of functional endothelium in the relaxant effect of *S. samarangense* extract, the endothelial layer was removed mechanically and considered denuded if Ach-induced relaxation was <10% [38].

The role of NO in *S. samarangense* extract-induced vasodilation was investigated in the presence of 100 µM of L-NAME (NOS inhibitor) [39]. The involvement of different receptors in the activation of eNOS was studied using 100 µM of atropine (muscarinic receptors blocker) [40] and 1 µM of propranolol (β-adrenoceptors blocker) [40]. The role of cyclases was studied using 1 mM of ODQ (sGC inhibitor) [41] and 30 µM of MDL (an adenylate cyclase (AC) inhibitor) [42]. The role of hyperpolarization was studied using high extracellular K^+^ by adding 30 mM of KCl (endothelium-derived hyperpolarizing factors (EDHF) inhibitor) [22,23] and 10 µM of indomethacin (a cyclooxygenase inhibitor) [43]. To study the role of the different potassium channels in the observed vasodilation of *S. samarangense* extract, the following blockers were used: 10 mM of TEA, a Ca^2+^-gated potassium channels blocker) [27], 1 mM of 4-AP (a voltage-dependant potassium channel blocker) [29], and 10 µM of glimepiride (an ATP-sensitive potassium channel blocker) [44]. All the blockers were introduced to the organ bath 30 min prior to PE addition.

The effect of extracellular Ca^2+^ on the vasodilation produced by *S. samarangense* extract was investigated as detailed in previous studies [34,45]. In brief, aorta sections with intact endothelium were kept for 30 min in normal Krebs buffer, and then the buffer was replaced with Ca^2+^ free Krebs buffer and kept for another 30 min. A single dose of 10 µM PE or 80 mM KCl was then added, and the response was recorded. Then after, the responses following adding raising concentrations of CaCl_2_ (1.25 to 10 mM) were recorded in absence or presence of *S. samarangense* extract (30 and 90 µg/mL).

### 4.5. Molecular Docking

The crystal structures of sGC receptor in the activated state (PDB ID: 7D9R) and in the inactivated state (PDB ID: 7D9T) were downloaded from pdb (www.rcsb.org). The compounds prevailed in *S. samarangense* extract were downloaded as SDF files from PubChem (https://pubchem.ncbi.nlm.nih.gov/). The chemical structures of the compounds were corrected by adding the hydrogen atoms, then energy was minimized, and their partial charges were assigned according to the physiological pH. The compounds’ structures were then compiled into one database that was used for docking. Molecular operating environment (MOE) software, 2020.0901 (Chemical Computing Group Inc.; Montreal, QC, Canada, H3A 2R7, 2022) was used to run the docking rounds. The docking protocol was validated by docking the co-crystallized compounds riociguat (a stimulator of sGC) and the haem-independent sGC activator; cinaciguat into the activated and the inactivated receptor’s states, respectively, applying the same docking protocol. The docked ligands showed optimum superimposition on the co-crystallized ligands in the binding site with Rmsd values less than 1.5.

### 4.6. Statistical Analysis

The study was performed using GraphPad Prism 6.0 (GraphPad, San Diego, CA, USA), and the mean ± standard error of mean was used to illustrate the findings. A *p* value of less than 0.05 was regarded as significant when comparing the vasodilating activity of various concentrations of *S. samarangense* extract statistically by the two-way ANOVA and the Bonferroni post-hoc tests, respectively.

## 5. Conclusions

The present work shows that the bioactive components of *S. samarangense* extract can synergistically induce vascular relaxation through endothelial dependent mechanisms, which involve the NO/cGMP pathway, PGI2/cAMP pathway, and hyperpolarization. In silico findings suggest that the major extract’s constituents have a good potential to act, more likely, as sGC receptor stimulators than activators. Further in vivo investigations are still required to affirm the possible mechanisms of action highlighted in this work.

## Figures and Tables

**Table 1:** Docking scores obtained upon docking the major constituents in *S. samarangense* extract into sGC receptors in the activated and inactivated states.

Compound	Docking Score (kcal/mol)	
	sGC Activated State (7D9R)	sGC Inactivated State (7D9T)
Myricitrin	−14.77	−11.74
Malaferin B	−13.32	−11.92
Xanthohumol	−13.30	Fail
Sinapic acid	−12.12	−10.50
4-Hydroxybenzoic acid glucoside	−11.48	−9.83
Sinensetin	−11.28	−11.09
Jaceosidin	−10.99	−8.63
Oleacein	−10.97	Fail
Rosmarinic acid	−10.67	−12.53
Myrigalon B	−10.63	−9.33
Oleuropein aglycone	−10.61	−10.27
Tangeretin	−10.53	−10.32
Gardenin B	−10.50	−10.10
Curcumin	−10.40	Fail
Tricin	−10.39	−9.35
Pinocembrin	−9.36	Fail
Syringaresinol	−9.36	Fail
Phloretin	−9.34	Fail
Cryptostrobin	−9.15	−7.92
Caffeic acid	−8.96	Fail
Umbelliferone	−8.62	Fail
Isorhamnetin	−8.26	−9.78
Myrigalone B	−8.08	Fail
2-Methoxy-2-phenylacetic acid	Fail	−6.83
Resveratrol	Fail	−7.03
Galangin	Fail	−8.65
Geraldone	Fail	−8.56
Myricetin	Fail	−9.47
Quercetin	Fail	−8.67
Riociguat (reference stimulator)	−11.45	-
Cinaciguat (reference activator)	-	−14.97

## Data Availability

Data is contained within the article.
